# CAR-T therapy dilemma and innovative design strategies for next generation

**DOI:** 10.1038/s41419-025-07454-x

**Published:** 2025-03-27

**Authors:** Zhiwei Wang, Peixian Li, Xiaoyu Zeng, Jing Guo, Cheng Zhang, Zusen Fan, Zhiwei Wang, Pingping Zhu, Zhenzhen Chen

**Affiliations:** 1 The First Affiliated Hospital of Henan University, 475004 Kaifeng, China; 2 School of Life Sciences, Zhengzhou University, 100 Kexue Road, Zhengzhou, 450001 China; 3 CAS Key Laboratory of Infection and Immunity, CAS Center for Excellence in Biomacromolecules, Institute of Biophysics, Chinese Academy of Sciences, 100101 Beijing, China; 4 University of Chinese Academy of Sciences, 100049 Beijing, China

**Keywords:** Tumour immunology, Tumour immunology

## Abstract

Chimeric antigen receptor (CAR)-T-cell therapy has shown remarkable curative effects on hematological tumors, driving the exponential growth in CAR-T-related research. Although CD19-targeting CAR-T-cell therapy has displayed remarkable promise in clinical trials, many obstacles are arising that limit its therapeutic efficacy in tumor immunotherapy. The “dilemma” of CAR-T cell-based tumor therapy includes lethal cytotoxicity, restricted trafficking, limited tumor infiltration, an immunosuppressive microenvironment, immune resistance and limited potency. The solution to CAR-T-cell therapy’s dilemma requires interdisciplinary strategies, including synthetic biology-based ON/OFF switch, bioinstructive scaffolds, nanomaterials, oncolytic viruses, CRISPR screening, intestinal microbiota and its metabolites. In this review, we will introduce and summarize these interdisciplinary-based innovative technologies for the next generation CAR-T-cell design and delivery to overcome the key barriers of current CAR-T cells.

## Facts

- CAR-T-cell therapy plays an important role in tumor treatment strategies.
- The “dilemma” of CAR-T-cell therapy includes lethal cytotoxicity, restricted trafficking, limited tumor infiltration, an immunosuppressive microenvironment, immune resistance and limited potency.
- The solution to CAR-T-cell therapy’s dilemma requires interdisciplinary strategies.

## Open questions

- What specific mechanisms do interdisciplinary strategies employ to overcome the “dilemma” of CAR-T cell therapy?
- Do interdisciplinary strategies have the potential for clinical application to benefit patients?
- What are the potential challenges associated with interdisciplinary CAR-T-cell therapy strategies?

## Introduction

**Fig. 1:**
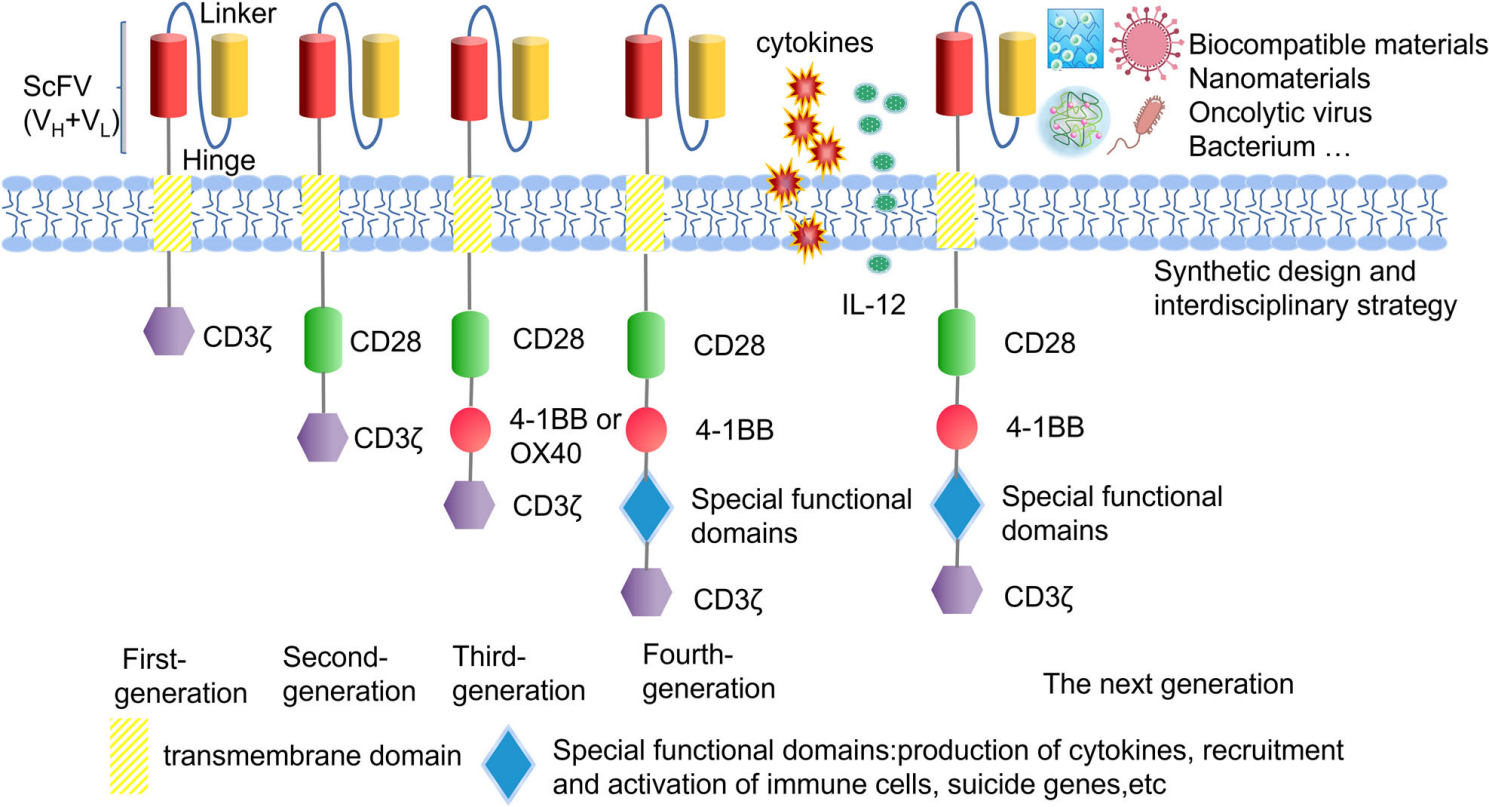
Schematic diagram of CAR structure. The first, second, third, fourth, and next generation CAR-T cells are shown. scFV single chain fragment variable, VH variable heavy chain, VL variable light chain.

CAR-T-cell therapy is the transfer of genetically engineered human T cells into the body of patients for disease treatment. CAR is a synthetic structure composed of three domains: (1) Extracellular domain containing the antigen recognition domain, which is usually a single-chain variable fragment (scFv) of antibody that recognizes tumor antigen [1]. (2) The transmembrane domain immobilizes CAR molecules on the cell membrane. (3) The intracellular domain, including the CD3ζ signaling domain and co-stimulatory domain, enhances T-cell proliferation, cytokine release and killing activity after antigen binding [2]. First-generation CARs contained only a CD3γ or CD3ζ signaling domain [3]. The addition of one or more costimulatory domains, such as CD28 or 4-1BB in the second and third generations, induced more cytokine production and promoted CAR-T cells proliferation [4–6]. Compared with CD28, CAR-T cells stimulated by 4-1BB have relatively mild expansion and longer survival time. To maximize their benefits, the fourth generation of CAR-T cells has been created by continuously exploring and improving the role of intracellular signaling domains [7–9] (Fig. 1).

In this review, we focus on the recent processes in CAR-T cells, especially interdisciplinary strategies to solve the dilemma of current CAR-T-cell therapy, including lethal cytotoxicity, restricted trafficking, limited tumor infiltration, immunosuppressive microenvironment and limited persistence.

## Lethal cytotoxicity

Lethal cytotoxicity is the primary concern limiting the widespread clinical use of CAR-T-cell therapy. Cytokine release syndrome (CRS) is a severe supraphysiological inflammatory response triggered by proinflammatory cytokines [10]. Currently, several therapies have been developed to avoid the lethal cytotoxicity, mainly divided into “self-control” and “active-control” therapies. Furthermore, “switch on” and “switch off” CARs have been constructed through a combination of “active-control” and other controllable tools, including suicide genes and sensors of light, sound or oxygen.

### Self-control

**Fig. 2:**
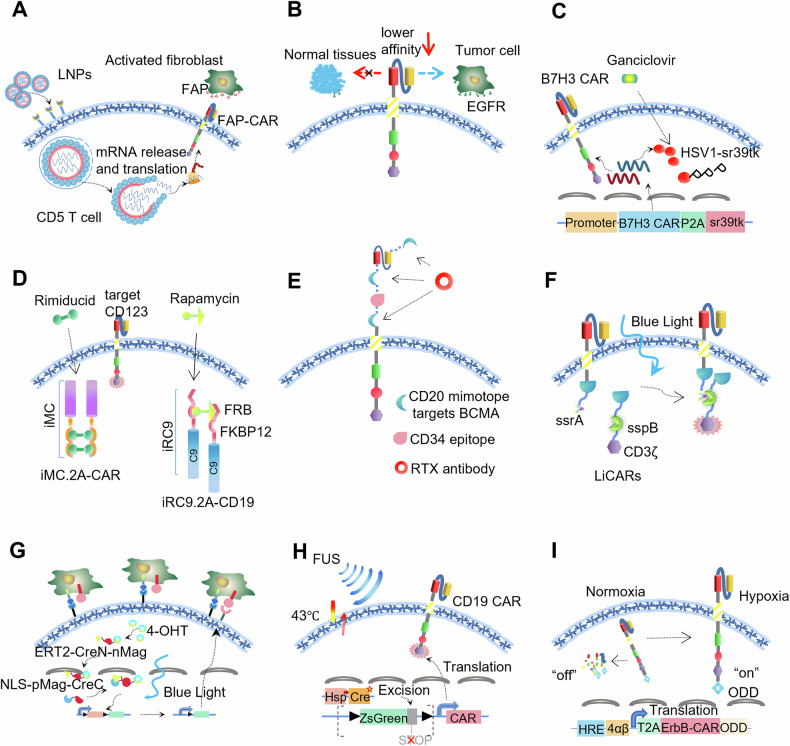
Molecular switches to control CAR-T cells. “Self-control” CAR-T design strategy, including CAR “expression control” such as CD5/LNP-FAP CAR-T cells (**A**) and CAR “affinity control” such as EGFRCAR-T cells (**B**). “Switch off active-control” CAR-T design strategy, including TK-ganciclovir suicide system such as B7H3-sr39tk CAR-T cells (**C**), iCasp9 suicide system such as iRC9 CAR-T cells (**D**), and RTX-CD20 suicide system such as CubiCAR-T-cell therapy (**E**). “Switch-on active-control” CAR-T design strategy, including light-induced activation such as LiCAR-T cells (**F**) and TamPA-Cre system (**G**), FUS-induced activation such as FUS-CAR-T cells (**H**), and hypoxia-induced activation such as HypoxiCAR-T cells (**I**).

“Self-control” reduces lethal cytotoxicity by limiting the expression or antigen affinity of CARs. One strategy to limit CAR expression is transient transfection of T cells with CAR-encoding messenger RNA (mRNA). Since CAR mRNA cannot be integrated into the genome, the number of CAR proteins expressed on the T-cell surface gradually decreases with the proliferation of T cells [11, 12]. CD5/LNP-FAP (FAP, a marker of activated fibroblasts)-CAR-T cell therapy was developed recently, in which modified FAPCAR mRNAs were packaged in CD5-targeting LNPs for transient FAPCAR expression (Fig. 2A). LNPs target and reprogram CD5 T cells in vivo to kill fibroblast cells for fibrosis reduction and successfully recover heart injury with limited toxicities because of the transient expression of FAPCAR, providing the possibility of editing T cells in vivo to generate tumor-targeting cells [13].

As the critical factor, the affinity of CAR is another layer for “self-control”. CAR-T cells with exceptionally high affinity can cause nonspecific targeting and serious cytotoxicity [14]. CAT CAR (a novel CD19 CAR) shows at least 40-fold lower affinity for CD19 than FMC63 CAR (a high-affinity CAR widely used in clinical research) but exhibits stronger antitumor activity and milder neurological side effects [15]. Similarly, tuning the affinity of CAR to EGFR (a TAA robustly expressed on multiple human cancers [16]) increases the ability of CAR-T cells to kill tumor cells rather than normal tissues with lower EGFR expression [17] (Fig. 2B). The strategy of reducing the binding affinity between CAR and its target antigen preserves CAR-T cells’ ability to kill high antigen-expressing cancer cells rather than healthy tissues with low antigen expression.

### Active-control—“switch off”

Rapid and flexible CAR-T cell elimination is an ideal strategy to protect patients from lethal cytotoxicity. Some suicide genes, such as herpes simplex virus-thymidine kinase (HSV-TK), inducible Caspase 9 (iCasp9) and CD20, have been utilized as safety switches to conditionally ablate CAR-T cells.

For osteosarcoma targeting, the HSV-TK gene was coexpressed with B7H3 (a TAA of osteosarcoma) to engineer B7H3-sr39tk CAR T cells [18]. B7H3-sr39tk CAR T cells showed powerful antitumor activity in vivo. The antiviral drug ganciclovir would bind to HSV-TK and inhibit CAR-T cell DNA replication. Then, a number of activated CAR T cells declined rapidly 2 days after ganciclovir administration, without damage to normal tissues (Fig. 2C). Rapamycin-induced/caspase-9-based (iRC9)-CD19 and MyD88 and CD40 (iMC)-CD123 are cotransfected to generate dual-switch (DS)-CAR-T cells, providing more choices to flexibly control CAR-T-cell activity (Fig. 2D) [19]. Rimiducid administration activated MyD88 and CD40 signaling and led a robust ligand-dependent induction of nuclear factor κB. DS-CAR-T cells were activated and expansion in a dose-dependent manner. As a typical iCasp9 cascade, iRC9 contains three elements: FKBP12, FRB and caspase-9 [20]. When the drug rapamycin is applied, the FKBP12-RAP-FRB ternary complex is formed, followed by dimerization and activation of caspase-9 to rapidly induce apoptosis of DS-CAR-T cells. Antibody-induced cell death is also used for CAR-T-cell elimination. CubiCAR is a trifunctional construct containing a human CD34 epitope (for cell enrichment), three CD20 mimotopes and CAR that targets BCMA^+^ tumor [21] (Fig. 2E). FDA-approved antibody rituximab (RTX) is a chimeric, monoclonal anti-CD20 antibody and CD20-expressing cells can be efficiently eliminated by RTX. CubiCAR specifically killed the BCMA^+^ human tumor cell line MM.1S and then could be efficiently depleted by RTX to minimize potential lethal cytotoxicity [22].

### Active-control—“switch on”

Optogenetics is a powerful tool for controlling the cytotoxicity of CAR-T cells. Light-switchable CAR T cells (LiCARs) “sleep” in darkness and do not function unless excited by blue light [23]. In this system, the functional domains of LiCARs are split into two halves. One domain employs bacterial peptide SsrA that is fused to the C-terminus of avena light-oxygen-voltage domain 2 (LOV2) and the other domain is conjected with binding partner SspB. The binding domain of SsrA is blocked by LOV2, which prevents it from binding to SspB. Therefore, anti-tumor therapeutic activity of LiCARs is blocked in the dark. When activated with blue light, the steric occlusion caused by LOV2 is unlocked, allowing the LOV2-SsrA and SspB to form optical dimerizer and activate LiCARs (Fig. 2F). Similarly, in a drug-photoactivatable “AND-gated” TamPA-Cre system, Cre recombinase is divided into two parts, CreN (2–59 aa) and CreC (60–343 aa, containing the nuclear location sequence), and then used for the construction of ERT2-CreN-nMag and NLS-pMag-CreC. CreERT2 serves as an additional security lock in addition to blue-light-inducible magnet protein (nMag, pMag) [24] (Fig. 2G). Collectively, CAR-T-cell activity is limited to the tumor site under the cooperation of tamoxifen and blue light, thus largely diminishing lethal cytotoxicity.

Focused ultrasound (FUS) can safely deliver mechanical energy into deep tissues for local heat generation, and has been widely used for adjuvant antitumor therapy [25]. FUS-CAR-T cells can be directly controlled with short-pulsed FUS stimulation [26]. CD19 CAR expression is blocked by the loxP-STOP-loxP element and is triggered by heat-shock-protein promoter-mediated Cre expression, which senses the heat generated by FUS (Fig. 2H). A total of 29% of FUS-CAR-T cells expressed CARs after a short FUS stimulation time and 82.9% of target tumor cells were eliminated by FUS-CAR-T cells.

Hypoxia-inducible factors (HIFs) respond to the low oxygen environment and result in tumor progression [27]. HypoxiCAR-T cells are designed based on the response of hypoxia-responsive elements (HREs) to HIF1α [28] (Fig. 2I). A cassette of 9 HRE repeats served as the CAR promoter; thus, many HypoxiCAR-expressing T cells were detected in the TME rather than normoxic tissues. To further limit off-tumor CAR T-cell activation, a 203-amino-acid oxygen-dependent degradation domain (ODD) is appended onto the C-terminus of CAR, leading to ubiquitination-dependent CAR protein degradation in normoxic tissues. This dual hypoxia-sensing system endows HypoxiCAR-T cells with features of hypoxia-restricted expression and activation of CAR in the TME and thus largely limits lethal cytotoxicity.

## Restricted trafficking and limited tumor infiltration

Trafficking and infiltration of T cells largely depend on the chemokines, whereas many solid tumors suppress the secretion of chemokines or secrete mismatched chemokines [29]. Moreover, solid tumors are substantially different from hematological tumors, and there is a more complex structure constituting a physical barrier to T-cell infiltration, containing abundant tumor-associated fibroblasts and blood vessels [30]. Here, we mainly review recent efforts to manipulate chemokines and overcome physical barriers to CAR-T infiltration.

### Chemokine-based CAR-T-cell enrichment

The activity of chemokine axes that are related to the CD8^+^ T-cell antitumor response generally determines the migration and infiltration capabilities of CAR-T cells. However, the activity of these chemokine axes is generally blocked in the TME, which typically prohibits the trafficking and infiltration [31]. Therefore, increasing the activity of antitumor chemokine axes serve as a promising strategy to enhance the antitumor ability of CAR-T cells (Table 1).

**Table 1:** Positive chemokine–receptor pathways of CAR-T cells trafficking and infiltration.

Chemokine-receptor	Mechanisms	Targets	Refs
CXCL9-CXCR3	CXCL9-expressing CAR-T cells attract more CAR-T cells to traffic and infiltrate tumors	Mesothelin	[36]
CXCL10-CXCR3	RIAD peptide blocks PKA localization and induces enhanced CXCR3/CXCL10-mediated migration rate of mesoCAR-RIAD T cells	Mesothelin	[33]
	Bevacizumab induces neuroblastoma cells to express high levels of CXCL10 and improves the tumor infiltration and antitumor efficacy of GD2-CAR T cells	GD2	[37]
	Lenvatinib upregulates expression of CXCL10 in tumor tissues, increasing CAIX-CAR-T cell infiltration	CAIX	[38]
CXCL11-CXCR3	Docetaxel upregulates CXCL11 expression in TME and subsequently enhanced the recruitment of HER2-CAR T cells	HER2	[39]
CXCL16-CXCR6	Forced expression of the CXCR6 enhances capacity of CAR-T cells to migrate towards CXCL16 producing pancreatic cancer cells	Mesothelin	[42]
CCL19-CCR7	Overexpressing CCL19 in CAR-T cells can attract additional CAR-T cells to the tumor	Mesothelin	[115]
		BCMA	[116]
CCR7-CCL21	CCL21 engineered CAR-T cells improve chemotaxis of CAR-T cells	CLDN18.2	[48]

*GD2* disialoganglioside, *CAIX* carbonic anhydrase IX, *HCC* hepatocellular carcinoma, *NSCLC* non-small cell lung cancer, *R/R MM* refractory/relapsed multiple myeloma, *BCMA* B-cell maturation antigen.

**Fig. 3:**
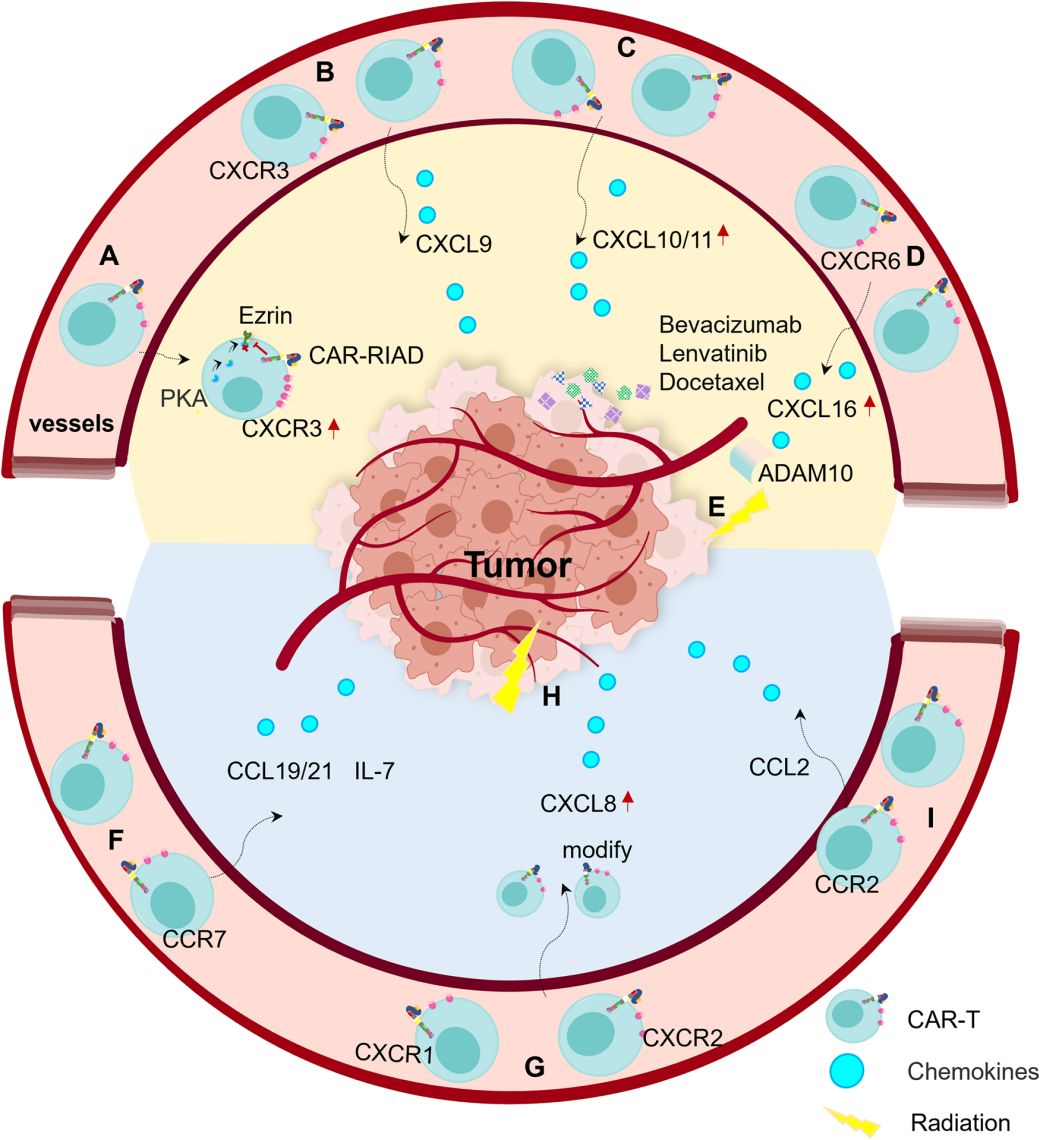
Chemokine-based CAR-T-cell enrichment. **A**–**C** CXCL9/10/11-CXCR3 axis. MesoCAR-RIAD T cells show enhanced intracellular CXCR3 expression (**A**). Intracellular CAR-T cell expressing CXCL9 (**B**) and drug-induced neuroblastoma-secreting CXCL9/10/11 (**C**) promote the recruitment and infiltration of CXCR3^+^CAR-T cells. **D**, **E** CXCL16-CXCR6 axis. Upregulated expression of CXCR6 (**D**) and CXCL16 secretion mediated by radiotherapy (**E**) increase the infiltration of CXCR6-expressing-CAR-T cells. **F** CCL19/CCL21-CCR7 axis. The ectopic expression of CCL19 or CCL21 recruits CD8^+^CCR7^+^CAR-T cells into the TME. **G**, **H** CXCL1/8-CXCR1/2 axis. Enforced expression of CXCR1 or CXCR2 in CAR-T cells (**G**), or ionizing radiation-induces CXCL8 expression (**H**), promote the infiltration of CAR-T cells. **I** CCL2/CCR2 axis. CCR2-bearing CAR-T cells harbor enhanced tumor infiltration and antitumor efficacy.

The CXCL9/10/11-CXCR3 axis mediates the migration and activation of CD8^+^ T cells and leads to positive clinical outcomes [32]. Type I protein kinase A (PKA) binds to membrane protein ezrin and negatively regulates the activity of CD8^+^ T cells, and peptide RIAD can bind to PKA with high affinity to abolish its negative regulation. With high expression of RIAD and CXCR3, MesoCAR-RIAD T cells show an enhanced trafficking and infiltration ability compared with mesoCAR T cells and exhibit enhanced tumor killing ability against mesothelin positive tumor cells (Fig. 3A) [33]. CXCL9 and CXCL10 recruit CD8^+^CXCR3^+^ T cells into the TME and result in long overall survival [34, 35]. Similarly, increasing the concentrations of CXCL9 and CXCL10 in the TME is an effective method to recruit CAR-T cells. For example, CXCL9-expressing CAR-T cells attract more CAR-T cells to traffic and infiltrate tumors (Fig. 3B) [36]. Interestingly, studies have demonstrated that the combination of targeted drugs (Bevacizumab, Lenvatinib and Docetaxel) and CAR-T-cell therapy enhances the concentrations of CXCL10 and CXCL11 in the TME and thus promotes the recruitment and infiltration of CAR-T cells to trigger a strong antitumor response (Fig. 3C) [37–39].

CXCR6 is the most relevant predictor of overall survival in human cancer patients [40]. CXCL16 is a ligand for CXCR6, which is preferentially expressed on tumor-infiltrating CD8^+^ T cells but absent on peripheral CD8^+^ T cells [41]. Upregulated expression of CXCR6 enhanced the ability of MSLN-CAR-T cells to migrate and infiltrate from the periphery into tumor tissues [42] (Fig. 3D). In a radiotherapy experiment for breast cancer, radiotherapy upregulated ADAM10 expression to mediate the release of CXCL16, and finally increased the infiltration of CD8^+^CXCR6^+^ T cells [43] (Fig. 3E). Therefore, the combination of CXCR6-expressing-CAR-T-cell therapy and radiotherapy may be a more effective treatment method.

CCR7 is mainly expressed on naïve and central memory T cells, and their activity is critical for the efficiency of CAR-T-cell therapy [44, 45]. Unfortunately, the CCL19/CCL21-CCR7 axis mainly recruits memory CD8^+^ T cells into the spleen and lymph nodes rather than the TME. Coexpression of IL-7, a cytokine that plays a critical role in the generation and maintenance of memory CD8^+^ T cells [46], and CCL19 increased the proportion of memory CD8^+^ CAR-T cells and attracted more CAR-T cells in in vivo antitumor experiments [47] (Fig. 3F). Similarly, the ectopic expression of CCL21, another ideal chemokine that recruits CD8^+^CCR7^+^CAR-T cells into the TME, and IL-7 enhanced the survival and infiltration of claudin18.2 (CLDN18.2, an overexpressed TAA)-CAR-T cells [48].

There are various protumorigenic chemokines in the TME, and these chemotactic gradients not only promote the propagation and metastasis of tumor cells, but also recruit immunosuppressive cells to suppress the antitumor activity of CAR-T cells [49]. Accordingly, ectopic expression of these protumorigenic chemokine receptors is a promising strategy to improve the trafficking and infiltration of CAR-T cells (Table 2).

**Table 2:** Ectopic expression of protumorigenic chemokine receptors.

Chemokine-receptor	Mechanisms	Targets	Refs
CXCL8-CXCR1/2	Co-expression of the matched chemokine receptor CXCR2 in CAR T-cells results in increased migration	Integrin αvβ6	[51]
	Hepatocellular carcinoma tumors express high level of CXCR2 ligands and CXCR2 expression endowed CAR-T cells with improved trafficking ability and enhanced tumor accumulation	Glypican-3	[117]
	Radiation-induced CKCL8 release from the tumor can enhance intratumoral CXCR1 or CXCR2 modified CAR-T cells trafficking	CD70	[52]
CCL2-CCR2	High concentration of CCL2 recruits CCR2 expressing CAR-T cells	Mesothelin	[53]

The CXCL1/8-CXCR1/2 axis mediates the progression of multiple tumors, and high levels of CXCL1 and CXCL8 in tumors have been shown to be correlated with tumor burden and poor prognosis [50]. For several solid tumors with high levels of CXCL8, including pancreatic, breast, and ovarian cancer cells, enforced expression of CXCR1 or CXCR2 in A20-28z CAR-T cells showed a higher infiltration proportion in the tumor core, with altered specificity or cytolytic activity [51] (Fig. 3G). Furthermore, CXCL8 expression can be induced vigorously by ionizing radiation (Fig. 3H). Therefore, the trafficking and infiltration ability of CXCR1 or CXCR2 overexpressing CAR-T cells can be further enhanced by radiotherapy [52].

CCL2 is overexpressed in tumor cells while its receptor CCR2 is exclusively expressed in immunosuppressive cells, conferring CCL2-CCR2 as an oncogenic axis. CCL2 is upregulated in some solid tumors, such as malignant pleural mesothelioma (MPM) and non-small cell lung carcinoma (NSCLC). For these tumors, high concentration of CCL2 can be used to recruit CCR2 expressing CAR-T cells. In the MPM mouse model, CCR2-transduced CAR-T cells show enhanced tumor infiltration and antitumor efficacy [53] (Fig. 3I). In the NSCLC xenograft model, tumors were completely ablated by infiltrating CCR2-modified CAR-T cells [54].

### Biocompatible materials to overcome physical barriers

Biocompatible materials have the capacity to continuously deliver activated live immune cells into tumors [55]. A variety of biocompatible materials have been used to overcome physical barriers and trafficking difficulties, and provide sustained, local release of CAR-T cells.

**Fig. 4:**
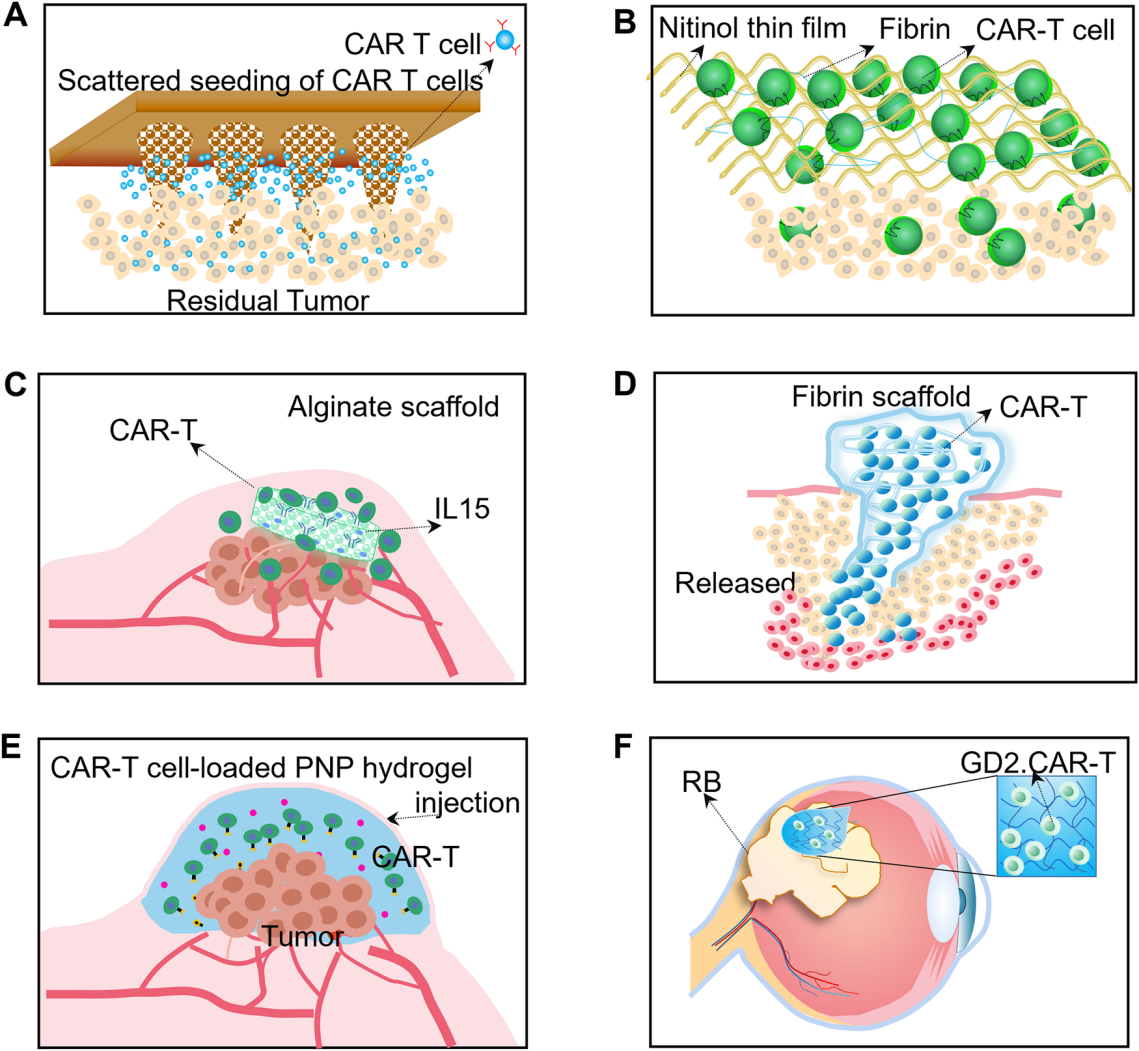
Biocompatible materials overcome physical barriers and trafficking difficulties. **A** PMN patch for CAR-T-cell scattered seeding and delivery. **B** TFN micromesh delivers plentiful and robust CAR-T cells. **C** Porous polysaccharide scaffold supports the rapid migration and sustained release of CAR-T cells. **D** Fibrin gel delivery system for overcoming the physical barriers of CAR-T cells. **E** Locally injectable CAR-T-cell-loaded PNP hydrogel. **F** PEG thermosensitive hydrogel for intraocular local injection of CAR-T cells.

Polymeric porous microneedle (PMN) adopts biocompatible poly lactic-co-glycolic acid (PLGA) as a microneedle scaffold and has a jagged and porous surface, and CAR-T cells are loaded into countless pores [56] (Fig. 4A). Therefore, PMN patches can accommodate and allow the scattered seeding of CAR-T cells intratumorally. Compared with the traditional intratumoral injection method, the PMN strategy effectively increases the breadth and depth of CAR-T cell distribution in an orthotopic pancreatic tumor model and “airborne force” CAR-T cells continuously restrain tumor growth.

For CAR-T-cell delivery and activation, the Matthias Stephan group designed a 10-micron-thick metal film, which is made of nickel-titanium alloy that can be safely implanted in the body with excellent biocompatibility. Thin film nitinol (TFN) micromeshes are coated with anti-CD3, anti-CD28 and anti-CD137 antibodies to recruit and activate tumor specific CAR-T cells. After implantation in a mouse model of unresectable ovarian cancer, TFN micromeshes recruit plentiful and robust CAR-T cells to the tumor. Tumors in all mice were disappeared at 10 days and 70% mice remained tumor-free at 20 days [57] (Fig. 4B).

Macroporous scaffolds are made of modified alginate and have a high-capacity encapsulation of CAR-T cells, IL-15 superagonists, anti-CD3 antibodies, anti-CD28 antibodies, and anti-CD137 antibodies [58]. After implantation onto the tumor surface, activated CAR-T cells egress from implants along with the degradation of alginate, leading to significant tumor regression and improved survival in a multifocal ovarian cancer model (Fig. 4C) [59].

In addition to alginate, fibrin gels are also used to overcome physical barriers. Specifically, the blood-brain-barrier is the primary obstacle to delivering CAR-T cells to glioblastoma [60]. A new study developed a porous fibrin gel by enzymatic reaction of human fibrinogen and thrombin [61] (Fig. 4D). In an in vitro assay, the porous fibrin gels encapsulated and gradually released CAR-T cells without impairing their viability, propagation and functionality. In an in situ glioblastoma resection model, porous fibrin gel significantly delayed the tumor growth, with 64% cancer-free mice at 94 days while the percent was 20% in the CAR-T alone group.

Locally injectable hydrogels retain the antitumor function of tumor-specific T lymphocytes without invasive surgical implantation procedures [62]. Recently, polymer-nanoparticle (PNP) hydrogels were used to controllably deliver CAR-T cells for solid tumor treatment [63] (Fig. 4E). PNP hydrogels loaded with CAR-T cells can be delivered to solid tumors or their metastases by a simple-to-implement strategy, such as direct injection or catheter delivery. As result of their unique architecture, PNP hydrogels provide sufficient and sustained CAR-T-cell exposure to the entrapped cytokine IL-15, and thus drive the expansion and activity of CAR-T cells in both local infusion assays and metastatic models.

Retinoblastoma (RB) is a pediatric retinal tumor, and enucleation is usually the only option for advanced stage patients [64]. Ganglioside GD2 is the overexpressed TAA of RB [65]. CAR-T cells targeting GD2 have been proven to effectively kill RB tumor cells effectively [66]. Chitosan-polyethylene glycol (PEG) thermosensitive hydrogel is biocompatible and biodegradable and forms hydrogels after intraocular local injection and continuously releases CAR-T cells, endowing GD2 CAR-T cells with durable antitumor activity and finally effectively preventing tumor recurrence (Fig. 4F).

## Immunosuppressive microenvironment

A major obstacle for CAR-T-cell therapy is the immunosuppressive TME, including immunosuppressive checkpoint ligands/receptors, immunosuppressive cells and cytokines [67, 68]. Many strategies have been designed to enhance CAR-T-cell function in the TME. Here, we focus on nanomaterials and oncolytic viruses, which provide new strategies to overcome the immunosuppressive TME barrier.

### Nanomaterials

**Fig. 5:**
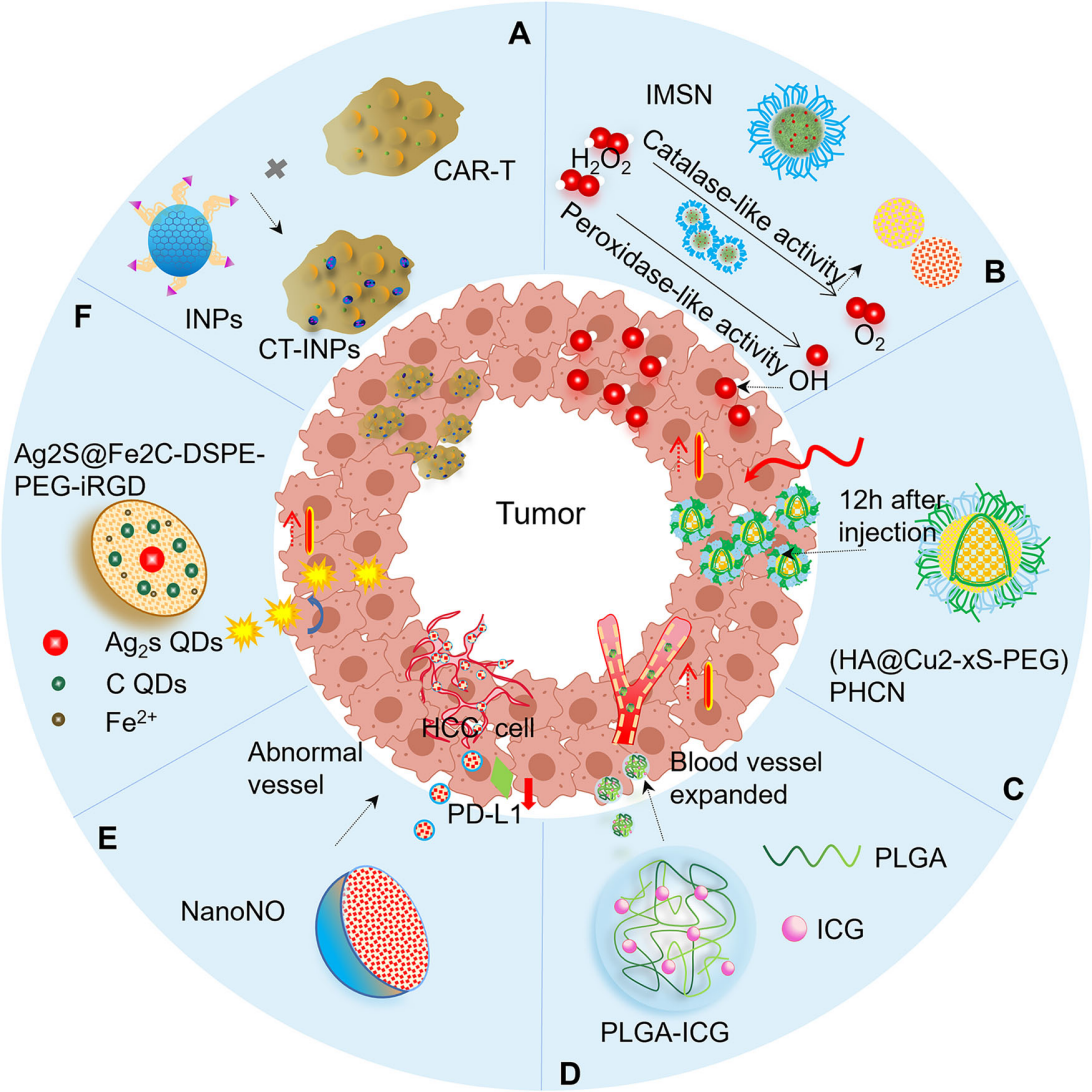
Nanomaterials modulate the immunosuppressive TME. **A** CT-INPs mildly elevate intratumoral temperature and modulate immunosuppressive microenvironment. **B**, **C** Nanozymes with high and stable catalytic properties for TME remodeling. IMSN with peroxidase-like and catalase-like activities (**B**) and PHCN with excellent stability and photothermal-nanocatalytic properties (**C**) remodel the immunosuppressive TME. Nanomedicines normalize the abnormal tumor blood vessels, such as PLGA-ICG nanoparticles (**D**), NanoNO (**E**) and Ag2S@Fe2C-DSPE-PEG-iRGD (**F**).

Proliferating tumor cells are sensitive to heat, and moderate temperatures (43–50 °C) that normal cells can tolerate are lethal to tumor cells [69]. Taking advantage of indocyanine green (ICG), an FDA-approved photothermal therapy (PTT) agent that produces mild photothermal heating by NIR laser irradiation, Chen et al. generated ICG nanoparticles (INPs), which are nanophotosensitizers with an excellent photothermal effect that can be used to modulate the immunosuppressive microenvironment [70] (Fig. 5A). In a mouse model, INP-engineered CAR-T biohybrids (CT-INPs) extensively aggregated at the tumor margin and penetrated deeply into the tumor 6 h after injection, inducing the intratumoral temperature to 43–44 °C after 15 min of laser treatment. This mild temperature is tolerated by normal cells but is sufficient to eradicate tumor cells and contributes to TME reconstitution, in which more infiltrating CAR-T cells, monocytes and NK cells are observed.

Nanozymes have been widely explored to regulate the TME due to their high and stable enzyme-like catalytic characteristics. Xu et al. constructed iron manganese silicate nanoparticles (IMSN), a nanozyme with peroxidase-like and catalase-like activities [71] (Fig. 5B). Under acidic conditions, IMSN degrades H_2_O_2_ into hydroxyl radicals and oxygen. Hydroxyl radicals have high cytotoxicity to tumor cells and oxygen creates a favorable environment for immune cells, especially T lymphocytes. The HA@Cu2-xS-PEG (PHCN) nanozyme exhibits excellent stability and photothermal-nanocatalytic (PNC) properties to confine enzyme-like catalysis in TME [72] (Fig. 5C). Twelve hours after injection, PHCN was enriched in the tumor and the tumor temperature rapidly increased to 51.97 °C under NIR laser irradiation. In NSCLC models, PHCN remodels the immunosuppressive TME with excellent PNC effects and improves overall survival compared to B7-H3 CAR-based therapy alone.

In addition to the features of low pH, hypoxia and immunosuppression, abnormal neovessels in tumors set up a chaotic maze for CAR-T cells [73]. To promote the intratumoral recruitment of CAR-T cells, ICG is encapsulated by PLGA to form PLGA-ICG nanoparticles, which increase the intratumoral temperature to induce the dilation of tumor vasculatures (Fig. 5D). PLGA-ICG nanoparticles reduces the compact structure of the tumor, and expands tumor blood vessels to increase blood perfusion. Consequently, increased numbers of monocytes, DCs and CAR-T cells are detected in the TME [74]. Taking advantage of NO, an important regulator of angiogenesis and functional maintenance, Sung et al. developed a nanodelivery system NanoNO, which packages and delivers NO to tumor blood vessels to promote their normalization [75] (Fig. 5E). NanoNO-induced blood vessel normalization further reduces PD-L1 expression, inhibits the transformation of TAMs to immunosuppressive M2 type and increases T-cell infiltration in HCC models. The collaboration of nanozymes and tumor vascular normalization therapy “unlocks” the breast cancer TME [76] (Fig. 5F). These “proof of concept” works reveal the potential of tumor blood vessel normalization in TME modulation for CAR-T-cell therapy.

### Oncolytic viruses

**Fig. 6:**
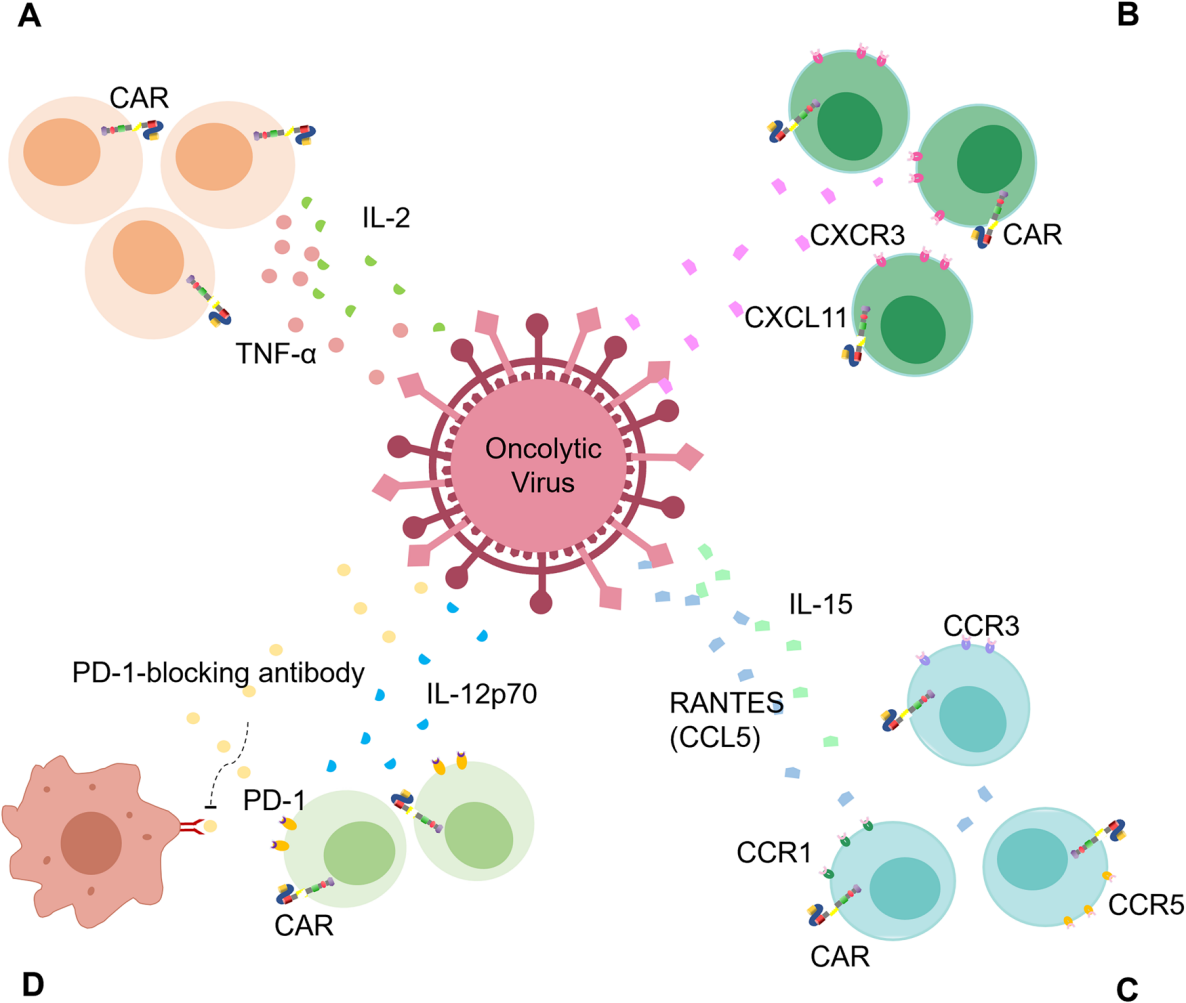
Cytokine-delivering OVs modulate the immunosuppressive TME. **A** OAd-TNF-α-IL2 delivers the pro-T-cell cytokines IL-2 and TNF-α to the immunosuppressive TME. **B** VV.CXCL11 augments antitumor efficacy of CAR-T cells via supplementation with CXCL11. **C** Ad5Δ24.RANTES.IL-15 OVs are equipped with both RANTES and IL-15. **D** CAd12-PDL1 blocks PD-L1 with its blocking antibody and prevents the loss of CAR-T cells with IL-12p70.

Many researches have demonstrated that cytokine-secreting Oncolytic viruses (OVs) can “reprogram” the TME and effectively induce antitumor immune responses [77]. OAd-TNF-α-IL2 delivers the pro-T-cell cytokines IL-2 and TNF-α in the immunosuppressive TME of pancreatic cancer, and enhances the antitumor efficacy of mesothelin-CAR T-cell therapy [78] (Fig. 6A). Likewise, the expression landscape of chemokines within the TME diametrically impacts the efficacy of CAR-T cells, and some oncolytic vaccinia viruses have been engineered to supplement chemokines. For example, CXCL11 oncolytic vaccinia virus (VV. CXCL11) augments the antitumor efficacy of CAR-T cells via supplementation with CXCL11, a ligand for CXCR3 that is highly expressed in effector T cells [79] (Fig. 6B). However, in addition to the activation of effector T cells, supplementation of cytokines in the TME may also enhance the activity of immunosuppressive cells to suppress the antitumor response. For example, Ad5Δ24.RANTES.IL-15 OVs are equipped with both RANTES and IL-15 [80] (Fig. 6C). IL-15 inhibits apoptosis and promotes proliferation of effector T cells. RANTES is a chemokine that is conjugated with multiple T-cell CCR receptors, so many T-cell subsets are recruited to TME, including CAR-T cells [81]. Collectively, Ad5Δ24.RANTES.IL-15 OVs possess a stronger ability to magnify the antitumor activity of CAR-T cells.

Immunosuppressive checkpoint ligands are important inhibitors for CAR-T cells in TME. OVs equipped with PD-L1-blocking mini-antibodies can help CAR-T cells break down the TME barrier. Shaw et al. constructed CAd12-PDL1 to express PD-L1-blocking antibody and IL-12p70, a cytokine with inhibitory effect on the death of CAR-HER2 T cells, and the demonstrated that combination of CAd12-PDL1 and CAR-HER2 T cells inhibits the propagation of both primary and metastasized tumors [82] (Fig. 6D).

### CAR-T-derived exosomes

Exosomes are nanoscale extracellular vesicles, which contain bilayer lipid membrane and vesicle contents [83]. Exosomes inherit most of the characteristics of the parent cells and participate in intercellular communication. Studies have shown that CAR-T cells release a large number of exosomes after being stimulated by tumor antigens [84]. Unlike live CAR-T cells, antitumor function of CAR-T-derived exosomes would not be limited by the immunosuppressive microenvironment and thus exhibit sustained viability [85]. CAR-T-derived exosomes carry the CAR on their surface and package perforin/granzymes inside. These characteristics endow them the abilities to recognize and kill tumor cells and have been confirmed in preclinical experiments. HER2 CAR-T-derived exosomes shared multiple cytokines with their parental CAR-T cells, such as granzyme B, perforin, IL-17, IL-2, etc. [86]. HER2 CAR-T-derived exosomes preserved CAR-T functions, allowing them bound and penetrated specifically into HER-2 expressing target cells. In another study, MSLN-CAR-T cell-derived exosomes achieved 52%–66% tumor growth inhibition and showed dose-dependent tumor growth inhibition [87]. Moreover, leakage of PD-1 expression on CAR-T-derived exosomes avoids the immunosuppression of the PD-1 pathway [84].

Nanoscale CAR-T cell-derived exosomes exhibit good biocompatibility and can deliver small molecules to target cells. Xu et al. loaded the CAR-T-derived exosomes with MYC-targeting sgRNA/Cas9 plasmids and successfully inhibited tumor growth [88]. In a recent study, chemotherapeutic drug paclitaxel was encapsulated in CAR-T-derived exosomes and administered to lung cancer mouse model. Theses engineered exosomes efficiently distributed to the tumor and led to rapid tumor ablation [89]. In another study, CAR-T cell-derived exosomes were equipped with both paclitaxel and anti-PD-L1 scFv to produce a hybrid nanovesicle called LipCExo@PTX [90]. This combination therapy reversed the immunosuppressive microenvironment and further enhanced the anti-tumor effect of CAR-T-derived exosomes. In addition to anti-tumor chemotherapeutic drugs, RNA vaccines offer another alternative strategy to enhance the anti-tumor effect of CAR-T-derived exosomes. In addition to anti-tumor chemotherapy drugs, RNA vaccines provide another alternative strategy to enhance the anti-tumor effect of exosomes. RN7SL1 is an endogenous RNA that activates RIG-I/MDA5 signaling and promotes CAR-T cells expansion and differentiation. Lexus et al. loaded RN7SL1 into CAR-T-derived exosomes to improves autonomous CAR-T cell function [91]. Interestingly, RN7SL1-loaded exosomes selectively accumulated in endogenous intratumor endogenous immune cells in addition to CAR-T cells. Thus, RN7SL1 delivery reversed the immunosuppressive microenvironment and synergistically reduce tumor burden. Overall, CAR-T-derived exosomes is potent strategy to overcome clinical barriers of CAR-T cell therapy.

## Limited potency and persistence

The complicated TME limits the potency and persistence of CAR-T cells [92]. In this chapter, we mainly discuss the contribution of two successful superstars, clustered randomly interspersed short palindromic repeats (CRISPR) screening and intestinal microbiota, in improving the potency and persistence of CAR-T cells.

### CRISPR-screening

**Fig. 7:**
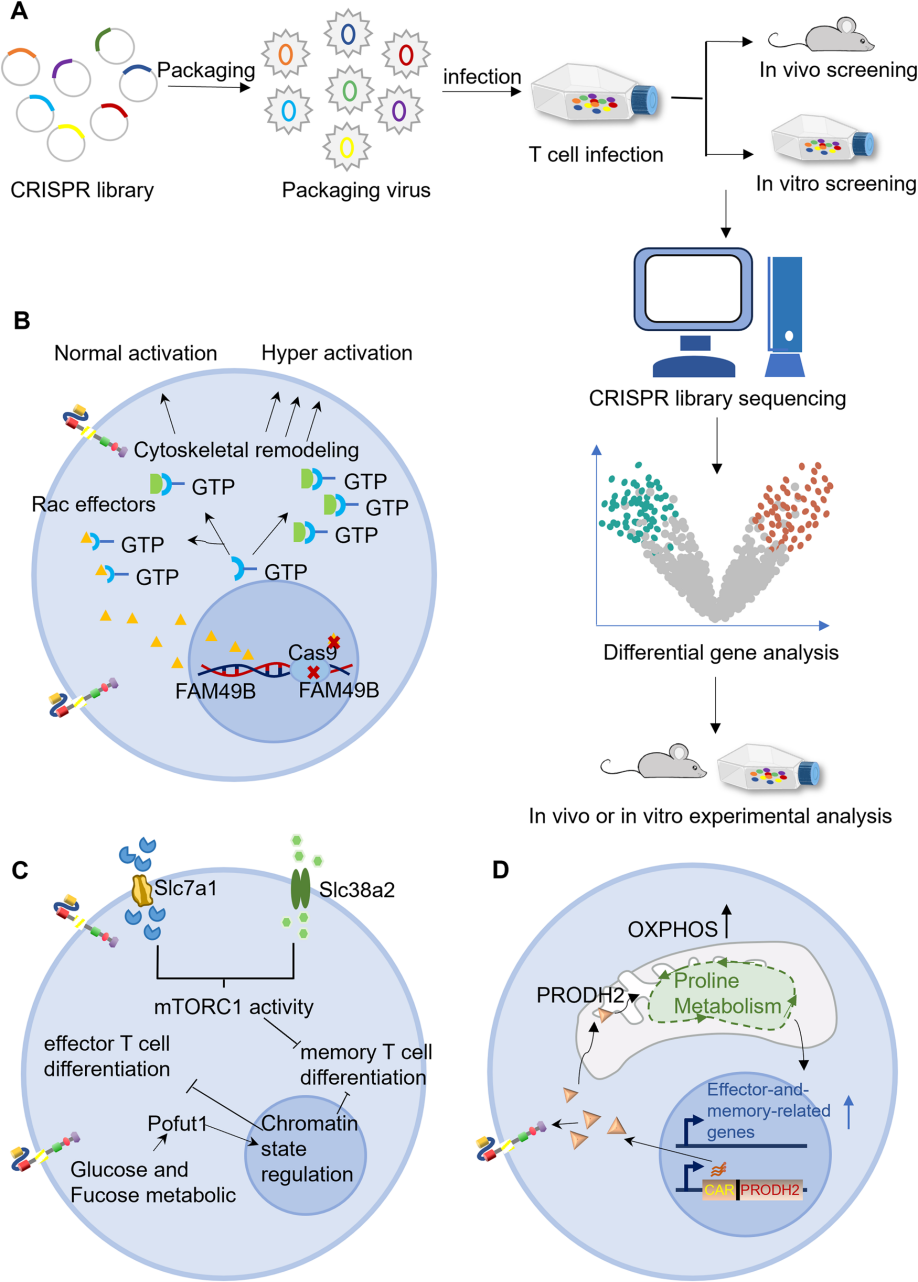
CRISPR screening strategy for improving potency and persistence of CAR-T cells. **A** CRISPR screening pipeline. **B** Genome-scale CRISPR screening defines FAM49B as negative regulator in T cell activation. **C** Metabolism-related CRISPR-screening reveals that Slc7a1 and Slc38a2 inhibit the differentiation of effector memory T cells and Pofut1 impairs the activation of T_MEM_ and T_EFF_ cells. **D** Genome-scale CRISPRa screening revealed the critical role of Praodh2-reshaped proline metabolism in CAR-T-cell therapy.

CRISPR-Cas9-based genome-editing tools have been successfully applied in cancer immunotherapy [93] (Fig. 7A). Negative immune regulators limit the potency and persistence of T cells. To identify negative regulators of T cells, Shang et al. performed genome-scale CRISPR screening with human Jurkat T cells [94]. FAM49B, an uncharacterized gene, is top-ranked in the negative regulator list. FAM49B inhibits the activation of T cells via actin polymerization, which may be a promising strategy to enhance the potency and prolong the persistence of CAR-T-cell therapy (Fig. 7B). Given the complexity of the TME, in vivo screening has the advantage of identifying key regulators in a true physiological environment. In a genome-scale CD8^+^ T screening in vivo, DHX37, a highly conserved DEAH box RNA helicase, hit a potential negative regulator of T-cell degranulation [95]. Because of the lack of safe DHX37-targeting compounds, genetic editing of DHX37 in CAR-T cells provides a potential way to increase the potency of CAR-T cells.

The metabolic activity of T cells determines their antitumor function [96]. Through CRISPR-screening for metabolism-related genes, the Hongbo Chi group revealed that some amino acid transporters negatively regulate the cytotoxic function of CD8^+^ T cells. Deletion of Slc7a1 and Slc38a2, two amino acid transporters, inhibited mTORC1 activity, increased persistence and decreased death of effector memory T cells (T_MEM_) [97] (Fig. 7C). Besides Slc7a1 and Slc38a2, protein-O-fucosyltransferase-1 (Pofut1) was also screened as another negative regulator of the immune response. Pofut1 simultaneously limits the activities of memory T cells (T_MEM_) and effector T cells (T_EFF_), whose function is a prerequisite for antitumor effects [98]. The Chi group also revealed that Pofut1 impairs T_MEM_ and T_EFF_ by regulating chromatin and metabolic states (Fig. 7C). Interestingly, The Cancer Genome Atlas (TCGA) database confirms that Pofut1 is negatively correlated with the survival of cancer patients treated with immunotherapies. Therefore, CRISPR screening of metabolism-related genes provides implications for CAR-T-cell therapy, and another study provides more direct evidence for this view [99]. In this research, abrogation of Regnase-1 facilitates the expression of naïve/memory-related genes and prolongs the lifespan of OT-I-Cas9 CD8^+^ T cells. Similarly, Regnase-1-null CD19-CAR-T cells show enhanced tumor elimination and prolonged survival in tumor-bearing mice. Besides, genome-scale CRISPRa screening revealed the critical role of Praodh2-reshaped proline metabolism in CAR-T-cell therapy [100] (Fig. 7D). Praodh2 overexpression significantly enhanced the expression levels of T cell activation markers and inhibited the Cleaved Caspase-3 expression, eventually leading to stronger cytolytic activity of CD22-CAR, BCMA-CAR and HER2-CAR T cells.

### Microbiota and their derived metabolites

**Fig. 8:**
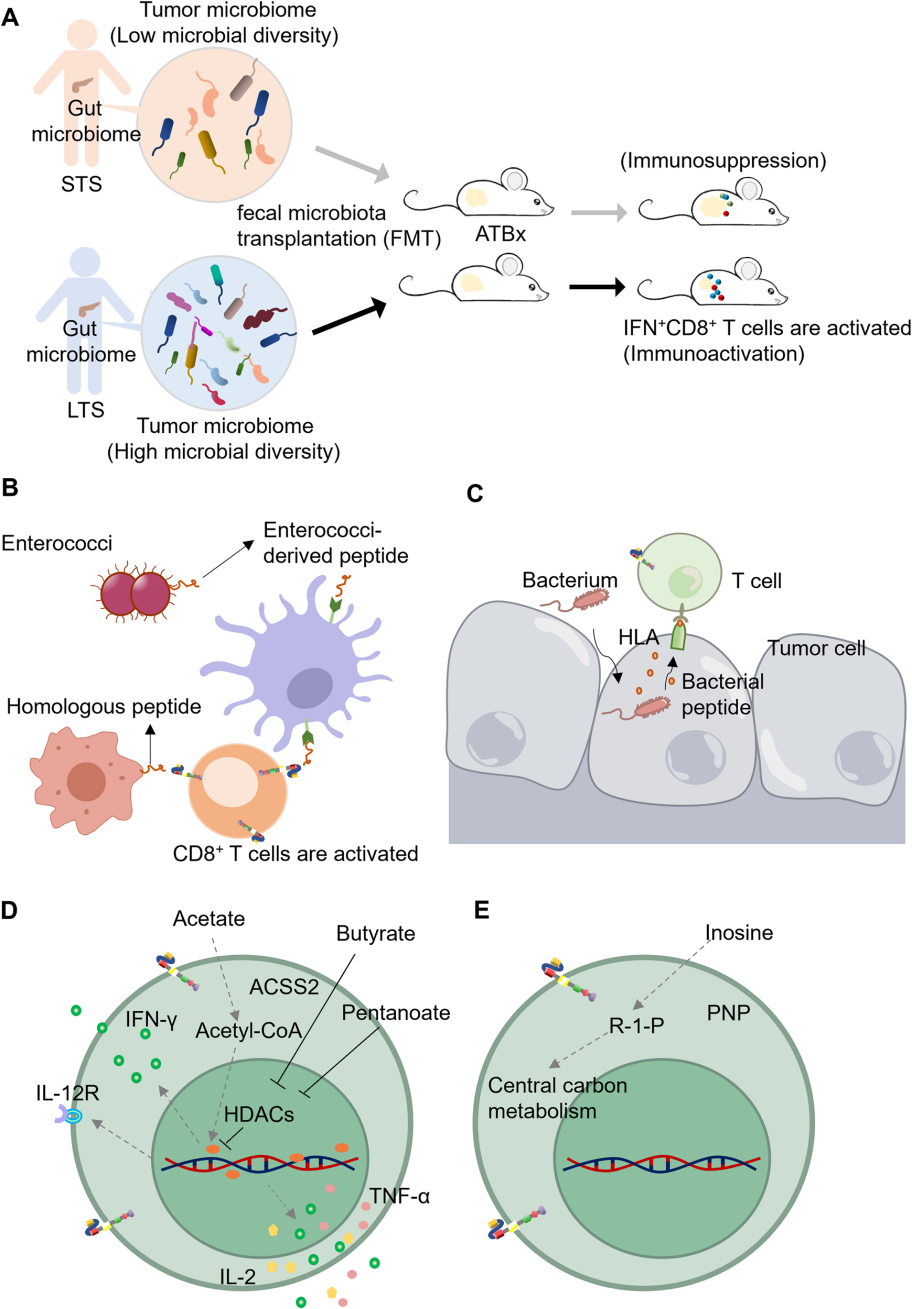
Microbiota and their metabolites in CAR-T-cell therapy. **A** The diversity of microbiome, including gut microbiome and tumor microbiome, has an indispensable impact in T cell activation. **B** Bacterial peptides homologous to tumor peptides induce CD8^+^ T-cell activation for tumor targeting. **C** Microbiota enter tumor cells, and bacterial peptides are presented by the HLA molecules of tumor cells. **D** Microbiota-derived SCFAs enhance the potency of CD8^+^ T cells via regulating metabolism and acetylation levels. **E** Microbiota-derived inosine serves as a carbon source to activate CD8^+^ T cell.

Given the close relationship between intestinal microbiota and tumor immunotherapy, a retrospective study assessed the clinical information of 228 patients who received CD19 CAR T cells [101]. The study found that decreased alpha-diversity (defined as the number and distribution of organisms) is associated with the immunotherapeutic response [102], indicating the association between the diversity of intestinal microbiota and the response of CAR-T therapy. One study demonstrated that gut microbiota shared 25% common composition between the matched tumor microbiota, while there was no detectable microbiota in adjacent normal tissues. Through a fecal microbial transplantation assay, bacteria derived from the intestine and tumor of long-term survivors were shown to significantly activate IFN^+^CD8^+^ T cells and reduce tumor volume in a CD8^+^ T-cell manner (Fig. 8A). These studies provide evidences of intestinal microbiota modulating the immune system and enhancing the potency of CAR-T therapy.

An alternative mechanism for microbiota-induced CD8^+^ T-cell activation is the cross reactivity between bacterial antigens and tumor antigens, which means that some peptides derived from bacterial antigens are similar to peptides derived from tumor antigens [103]. The TMP1 peptide derived from enterococci (a type of intestinal bacteria) shares a strong homology with a peptide of proteasome subunit beta type-4 (PSMB4, a tumor antigen), and this homology arouses the bacteria-dependent antitumor activity of CD8^+^ T cells in an HLA-restricted manner (Fig. 8B). Interestingly, microbiota enter melanoma cells, and bacterial peptides are likely presented by the HLA molecules of melanoma cells. For example, 283 unique HLA peptides, which are derived from bacteria and presented by melanoma cells, have been identified and are expected to initiate the immune response [104] (Fig. 8C).

Accumulating evidences indicate that gut microbiota-derived short-chain fatty acids (SCFAs), such as acetate, butyrate, and pentanoate, have a positive effect on CAR T cells [105, 106]. As one of the major gut microbial metabolites, acetate also serves as a substrate for acetyl-CoA production and promotes IFN-γ expression in T cells [107]. Under glucose starvation conditions, acetate supplementation restores the effector function of T cells through ACSS2, which converts exogenous acetate into acetyl-CoA to meet the demand for glucose [108] (Fig. 8D). Acetyl-CoA, in turn, promotes histone acetylation and drives cytokine production in effector T cells. Butyrate is another gut microbiota-derived SCFAs that plays an immunoregulatory role [109]. Butyrate-treated CD8^+^ T cells exhibit impaired activity of histone deacetylases (HDACs), and this epigenetic alteration activates the ID2-dependent IL-12 pathway, finally promoting the proliferation and potency of CD8^+^ T cells (Fig. 8D). Similarly, pentanoate, another kind of SCFA secreted by *M. massiliensis*, strongly inhibits the activity of HDACs through the glycolytic metabolic pathway and stimulates the secretion of IFN-γ and TNF-α by CD8^+^ T cells [110] (Fig. 8D). Collectively, gut microbiota-derived SCFAs promote gene expression related to CD8^+^ T-cell survival, activation and persistence.

Inosine is not only a normal metabolite of the human body, but also a metabolite of the intestinal microbiota. The metabolic stress caused by the high nutrition demands of tumor cells restricts the potency and persistence of CAR-T cells. Inosine could replace glucose to provide nutritional support for CAR-T cells and restore potency, enhance the tumor elimination ability of GD2-CAR T cells and prolong the survival of tumor-bearing mice (Fig. 8E) [111]. Therefore, it offers a promising solution for CAR-T cells to supply microbial strains such as *B. pseudolongum* for inosine production or oral inosine.

## Conclusion and perspective

CAR-T-cell therapy has revolutionized the treatment of hematological tumors, making it one of the major breakthroughs in tumor therapy during the past decade. However, there are still some obstacles hindering CAR-T cells from curing more tumor patients. In this review, we summarized recent efforts to solve these obstacles, especially focusing on interdisciplinary strategies including synthetic biology-based ON/OFF switching, bioinstructive scaffolds, nanomaterials, OVs, CRISPR screening, intestinal microbiota and its metabolites. Fortunately, some of the strategies have already been applied in clinical studies (Table 3). Five studies aimed at resolving the lethal cytotoxicity issue of CAR-T cell therapy have progressed into clinical trials (NCT01355965, NCT01897415, NCT02443831, NCT00423124, NCT04196413). In the published data from low-affinity CD19 CAR clinical trial (NCT02443831), toxicity levels remained low, with no cases of severe CRS reported. The 1-year overall survival rate was 63% and the 1-year event-free survival rate was 46%. As for the barriers of restricted trafficking and limited tumor infiltration, clinical trials of CXCR2 (NCT05353530), CXCR4 (NCT04727008) or CXCR5 (NCT05060796, NCT04153799) ectopic expression CAR-T cell therapies are currently under investigations in Phase I.

**Table 3:** Next generation clinical CAR-T therapy.

Challenge	Strategies	Targets	Design	Identifiers
Lethal cytotoxicity	Self-control	Mesothelin	Transfecting T cells with mRNA encoding an anti-mesothelin CAR	NCT01355965, NCT01897415
		CD19	Tuning the affinity of CAR to CD19	NCT02443831
	Active-control	CD44v6	Coexpressing the suicide gene HSV-TK Mut2 and CAR CD44v6	NCT00423124
		GD2	Transducing T cells with 14g2a-CD8-BBz-iCasp9 retroviral vector expressing GD2-CAR	NCT04196413
Restricted trafficking and limited tumor infiltration	Chemokine-based CAR-T-cell enrichment	BCMA	Simultaneously expressing IL7 and CCL19	NCT03778346
		EGFR	CXCR5 modified EGFR-CAR-T cells	NCT05060796, NCT04153799
		BCMA	CXCR4 modified BCMA-CAR-T cells	NCT04727008
		CD70	CXCR2 modified CD70-CAR-T cells	NCT05353530
Immunosuppressive microenvironment	Oncolytic viruses	HER2	Combined injection of HER2-CAR-T cells and CAdVEC	NCT03740256
		Mesothelin	Combined injection mesothelin-CAR-T cells and VCN-01 (expressing hyaluronidase)	NCT05057715

Besides, the BCMA-7×19 CAR-T therapy clinical trial was initiated (NCT03778346). Two enrolled patients in the study responded effectively within 1 month and have experienced no relapse for over 1 year. In contrast, only two therapies that combine CAR-T cells with oncolytic viruses are currently undergoing Phase I trials (NCT03740256, NCT05057715). These interdisciplinary combination therapies may inspire more new therapeutic methods for CAR-T-cell therapy to overcome obstacles and thus warranted further clinical study.

In addition to external obstacles of CAR-T therapy, intrinsic limitations should also be considered in the future. A mathematical model demonstrated that patients’ T cells outcompete CAR-T cells. This competitive relationship put CAR-T cells at a disadvantage and limits their function [112]. Moreover, epigenetic profiling of CAR-T cells can determine clinical outcomes and predict which patients will benefit from CAR-T-cell therapy [113]. A 10-year follow-up report on CAR-T cell therapy shows that a small number of clones remain functionally active in patients 10 years after receiving CAR-T-cell therapy, demonstrating the existence of long-term CAR-T cells [114]. The intrinsic molecular characteristics of these long-term CAR-T-cell may provide new insights for CAR-T cells therapy. Therefore, a comprehensive understanding of the external and intrinsic features of CAR-T cells may lead to the development of a more promising therapy to overcome obstacles.
